# Exogenous Methyl Jasmonate Promotes Triterpene Accumulation in Loquat Callus

**DOI:** 10.3390/foods15061078

**Published:** 2026-03-19

**Authors:** Rui Zhang, Yongtao Liu, Jing Lin, Xiuping Chen, Weilin Wei, Jimou Jiang, Chaojun Deng, Shuning Li, Zhongqi Fan, Wenbing Su, Huijuan Wang

**Affiliations:** 1 Institute of Postharvest Technology of Agricultural Products, College of Food Science, Fujian Agriculture and Forestry University, Fuzhou 350002, China; 13011692657@163.com (R.Z.); 15608527529@163.com (Y.L.); 15396028003@163.com (J.L.); 15987237981@163.com (S.L.); fanzqi@fafu.edu.cn (Z.F.); 2 Fujian Breeding Engineering Technology Center for Longan and Loquat, Fruit Research Institute, Fujian Academy of Agricultural Science, Fuzhou 350013, China; cxp2516@126.com (X.C.); weiweilin01@163.com (W.W.); jjm2516@126.com (J.J.); dengchaojun2002@163.com (C.D.)

**Keywords:** loquat, triterpene metabolites, jasmonic acid (JA) signaling and JA response, *EjOSC*, *EjCYP716A*

## Abstract

Loquat (*Eriobotrya japonica* Lindl.) is a subtropical evergreen fruit tree that accumulates abundant bioactive triterpene compounds with diverse pharmaceutical activities. Its leaves have been used in traditional Chinese medicine for over 1000 years. Methyl jasmonate (MeJA) is a conserved elicitor that stimulates plant secondary metabolism. However, the regulatory mechanisms of terpenoid biosynthesis after MeJA treatment in loquat callus remain largely unknown. In this study, we employed an integrated targeted metabolomic and transcriptomic approach to investigate the effect of exogenous MeJA on terpenoid biosynthesis in loquat callus. In total, 131 terpenoid compounds were detected, including 112 triterpenes, six triterpene saponins, seven diterpenoids, three sesquiterpenoids and three monoterpenoids. After MeJA treatment, a total of 55 and 33 differential metabolites (DEMs) were identified at 24 h and 48 h, respectively. Most DEMs were triterpene compounds, displaying increased accumulation. Among them, ursolic acid showed the highest accumulation at 24 h, and betulinic acid was most abundant at 48 h. Meanwhile, transcriptome analysis showed significant upregulation of terpenoid biosynthesis genes, including *EjFPSs*, *EjSQEs*, *EjOSC2* and *EjCYP716A2*, as well as genes related to jasmonic acid (JA)-mediated signaling and JA-responsive genes in loquat callus treated with MeJA. Overall, these results provide a deeper understanding of the mechanism of terpenoid accumulation in loquat callus induced by MeJA and establish a theoretical basis for utilizing plant cell culture techniques to achieve production of the valuable terpenoid metabolites that are applied in the functional food and pharmacological industries.

## 1. Introduction

Loquat (*Eriobotrya japonica* Lindl.), belonging to the Rosaceae family, originates from southeast China. It is an economically important subtropical evergreen fruit tree [1]. Its fruits enrich various vitamins, soluble fiber, carotenoids and minerals [2]. Furthermore, its leaves also contain diverse biologically active constituents, such as triterpenes, sesquiterpenoid glycosides, flavonoids, phenylpropanoids and megastigmane derivatives [3,4]. Several of these compounds have been reported to possess antioxidative, anti-inflammatory, antitumor, antiviral and hypoglycemic activities [5,6]. Triterpenes, such as ursolic acid (UA) and oleanolic acid (OA), are the main class of bioactive compounds in loquat leaves, exhibiting significant pharmacological and nutraceutical potential. These compounds possess antioxidant activity, which makes them highly promising for developing functional foods and preserving food quality by inhibiting lipid oxidation. Forty-seven triterpenes have been isolated and characterized from leaves to date [7]. Moreover, the known diversity of loquat terpenoids is expanding with the advances in analytical methodologies and phytochemical investigation. Recently, a widely targeted LC-MS/MS metabolomics approach identified 193 terpenoid compounds in loquat flowers subjected to different processing treatments [8]. Despite these advances in terpenoid profiling across loquat tissues, the regulatory mechanisms underlying terpenoid biosynthesis, particularly in response to elicitors such as methyl jasmonate (MeJA), remain largely unexplored. In addition, the potential of loquat callus cultures as a controlled system for investigating MeJA-induced terpenoid accumulation has not been systematically investigated.

The biosynthesis of terpenoids in plants initiates with the formation of isopentenyl diphosphate (IPP) and its isomer dimethylallyl diphosphate (DMAPP) via either the cytosolic mevalonate pathway or the plastidial methylerythritol-4-phosphate pathway. These intermediates subsequently converted into different terpenoid classes, including monoterpenoids (C10), sesquiterpenoids (C15), diterpenoids (C20) and triterpenes (C30) [9,10]. For triterpenes biosynthesis, the first step is cyclizing 2,3-oxidosqualene catalyzed by oxidosqualene cyclases (OSCs) to generate diverse triterpene skeletons, such as α-amyrin, β-amyrin and lupeol [11,12]. Then, these precursors are further modified by cytochrome P450 monooxygenases (CYP450), UDP glucuronosyl transferases (UGTs) and acyltransferases (ATs), ultimately yielding structurally diverse triterpene compounds [9,13]. Previously, we had characterized key steps in loquat triterpene biosynthesis. Both EjOSC1 and EjOSC2 catalyzed the conversion of 2,3-oxidosqualene to a mixture of α-amyrin (AA) and β-amyrin (BA). Subsequently, EjCYP716A1 and EjCYP716A2 mediated the C-28 oxidation of AA and BA to produce UA and OA, respectively. Then, EjCYP716C1 and EjCYP716C2 catalyzed C-2α hydroxylation of OA and UA to form maslinic acid (MA) and corosolic acid (CA), respectively [14]. The identification of these key genes and their corresponding triterpene products can provide a foundation for investigating how exogenous elicitors such as MeJA regulate this biosynthetic pathway at both the transcriptional and metabolic levels through integrated metabolomic and transcriptomic analyses.

Plant callus cultures provide a platform for producing secondary metabolites under artificially controlled conditions without being affected by environmental factors [15]. This in vitro culture system offers a sustainable and scalable approach for producing valuable bioactive compounds that are utilized in the food and pharmacological industries [16]. Loquat callus tissue has been reported to accumulate substantial amounts of bioactive triterpenes [17,18]. MeJA, a plant hormone, serves as a conserved elicitor that can enhance the accumulation of secondary metabolites in plants [19]. Exogenous MeJA can induce the biosynthesis of major classes of secondary metabolites, such as isoflavonoid [20], terpenoids [21] and alkaloids [22], in plant cell cultures. This induced strategy has significant potential for improving the production of high-value food ingredients and nutraceutical compounds. For example, MeJA treatment increased the total flavonoids and phenolic compounds in finger millet (*Eleusine coracana* L.) sprouts following the elevated activity and expression of the key enzymes involved in flavonoid biosynthesis [23]. Similarly, MeJA-treated in vitro propagated shoots of *Catharanthus roseus* displayed a significant increase in alkaloids levels, as well as altered expression of regulatory and biosynthetic genes [24]. In callus cultures of *Lepechinia caulescens*, MeJA markedly increased the accumulation of triterpenes, including UA and OA [21]. However, the effects and underlying mechanisms of MeJA on triterpene production in loquat callus cultures remain largely unknown.

In this study, an integrated widely targeted metabolome and transcriptome analysis was performed to reveal the effects of MeJA on terpenoid accumulation in loquat callus cultures and identify the underlying molecular mechanisms. To the best of our knowledge, this is the first report characterizing MeJA-induced terpenoid accumulation in loquat callus, which provides a theoretical basis for developing plant callus culture strategies for sustainable production of valuable terpenoid metabolites with potential applications in functional foods and nutraceuticals.

## 2. Materials and Methods

### 2.1. Loquat Callus Culture Establishment and MeJA Treatments

Loquat (*Eriobotrya japonica* cv. Jiefangzhong) seeds were obtained from the National Germplasm Resource Nursery for Longan and Loquat, affiliated with the Fujian Academy of Agricultural Science (Fuzhou, China). Callus was induced from shoot tips, as previously described [18,25], with slight modifications. Seeds were sterilized with 70% (*v*/*v*) ethanol for 1 min, followed by rinsing three times with sterile distilled water. Subsequently, they were immersed in a solution of 50% (*v*/*v*) commercial bleach for 15 min, and then rinsed thoroughly with sterile water three times to completely remove all residual sterilant. Seeds were grown on the MS agar medium for one month to obtain loquat seedlings with leaves, stems and roots. These tissues were cut into small slices and placed on MS medium supplemented with 1-naphthaleneacetic acid (NAA) and 6-benzyladenine (BA) for callus induction at 25 °C. After three weeks of subculture, callus was treated with 200 µM MeJA with three biological replicates. Tissues were harvested at 0, 24 and 48 h, respectively, for further analyses.

### 2.2. Widely Targeted Metabolome Analysis in Loquat Callus Culture

Terpenoid metabolites were extracted from loquat callus as previously described, with minor modifications [26]. In brief, for each biological replicate, 20 mg of dried samples were independently soaked in 1 mL absolute ethanol for 2 h. Then, the samples were subjected to continuous ultrasonic extraction at 50 °C for 30 min, and this process was repeated twice. The combined supernatants were dried and redissolved in 1 mL methanol by vortexing for 15 min. After centrifugation at 12,000 rpm for 3 min at 4 °C, the supernatant was filtered and analyzed using an ultra-performance liquid chromatography (UPLC) system (UPLC, ExionLC™ AD; AB Sciex, Framingham, MA, USA) with an ESI-triple quadrupole-linear ion trap (QTRAP)-MS (AB Sciex, Framingham, MA, USA). The analytical conditions were as follows: UPLC separation was performed on an Agilent SB-C18 column (1.8 µm, 2.1 mm × 100 mm; Agilent Technologies, Santa Clara, CA, USA) at a flow rate of 0.35 mL/min with the column oven set to 40 °C. The mobile phase consisted of solvent A (0.1% formic acid in water) and solvent B (0.1% formic acid in acetonitrile). A linear gradient program was applied as follows: 0–9 min, 5–95% B; 9–10 min, 95% B; 10–11.1 min, 95–5% B; and 11.1–14 min, 5% B.

The qualitative analysis of terpenoid metabolites was performed using secondary mass spectrometry data information based on the self-built MWDB database (Wuhan, China). Terpenoid metabolites were quantified using multiple-reaction-monitoring mode (MRM) on a triple quadrupole mass spectrometer. After obtaining the mass spectrometry data of metabolites, peak area integration was performed for all detected compounds, and the peak areas of the same metabolite across different samples were normalized based on its chromatographic peaks. For quality control (QC) purposes, QC samples were prepared by pooling all individual samples and analyzed periodically throughout the analysis to monitor the stability and reproducibility of the system. Principal component analysis (PCA) was performed using statistics function prcomp within R to assess the variation between difference groups and samples within each group. Variable importance in projection (VIP) values of all metabolites from the OPLS-DA model were calculated to determine the relative importance of the metabolites. Differential metabolites were identified using variable importance in projection (VIP) ≥ 1 and a fold change of ≥1.20 or ≤0.83 [27].

### 2.3. RNA Isolation and Library Preparation

Total RNA was extracted using a commercial Plant RNA Kit (Tiangen, Beijing, China). After quality assessment, mRNA was captured from total RNA using oligo(dT)-attached magnetic beads, followed by the first- and second-strand cDNA synthesis. Sequencing libraries were then constructed using the NEBNext UltraTM RNA Library Prep Kit for Illumina (NEB, Ipswich, MA, USA), and library quality was evaluated using an Agilent 2100 analyzer (Agilent Technologies, Santa Clara, CA, USA) [28].

### 2.4. Transcriptome Sequencing, Sequence Assembly, Annotation and Differential Expression Analysis

Libraries were sequenced on an Illumina NovaSeq platform to generate paired-end reads. Clean reads were aligned to the ‘Jiefangzhong’ reference genome [14]. Gene annotation was performed using the previously reported databases [29]. Gene expression was estimated by FPKM (fragments per kilobase of transcript per million fragments mapped) [30]. Differentially expressed genes (DEGs) were identified using DESeq2 with false discovery rate (FDR) < 0.01 and fold change ≥ 2 [31].

### 2.5. Statistical Analysis

All experiments were performed with independent biological replicates. Data were expressed as mean ± standard deviation (SD). Figures were generated using GraphPad Prism 8.0 software. The correlations were calculated using biological replicates (*n* = 3) rather than averaged values. The Benjamini–Hochberg false discovery rate (FDR) correction was applied for multiple comparisons, and correlations with adjusted *p*-values < 0.05 were considered statistically significant.

## 3. Results

### 3.1. Widely Targeted Terpeneoid Profiling of Loquat Callus Under MeJA Treatment

**Figure 1:**
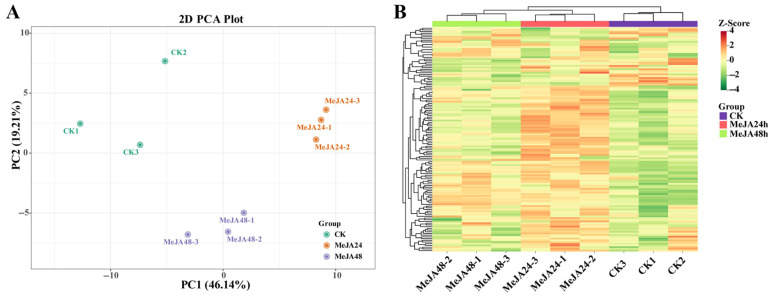
Multivariate statistical analysis of terpenoid metabolites in loquat callus. (**A**) Principal component analysis (PCA). (**B**) Hierarchical clustering analysis (HCA).

To evaluate the effect of MeJA on terpenoid accumulation in loquat callus, widely targeted metabolome profiling was performed by the UPLC-MS/MS method. In the principal component analysis (PCA) score plot, PC1 and PC2 were calculated to be 19.21% and 46.14%, respectively. The three experimental groups (CK, MeJA_24 h and MeJA_48 h) are distinctly separate, with three biological replicates of each group clustering together, demonstrating excellent experimental reproducibility and reliability (Figure 1A). Furthermore, the samples are clearly separated into three clusters on the heatmap (Figure 1B), suggesting notable differences in metabolite composition and abundances among three groups. These results suggested that MeJA treatment substantially changed the terpenoid profiling of loquat callus.

**Figure 2:**
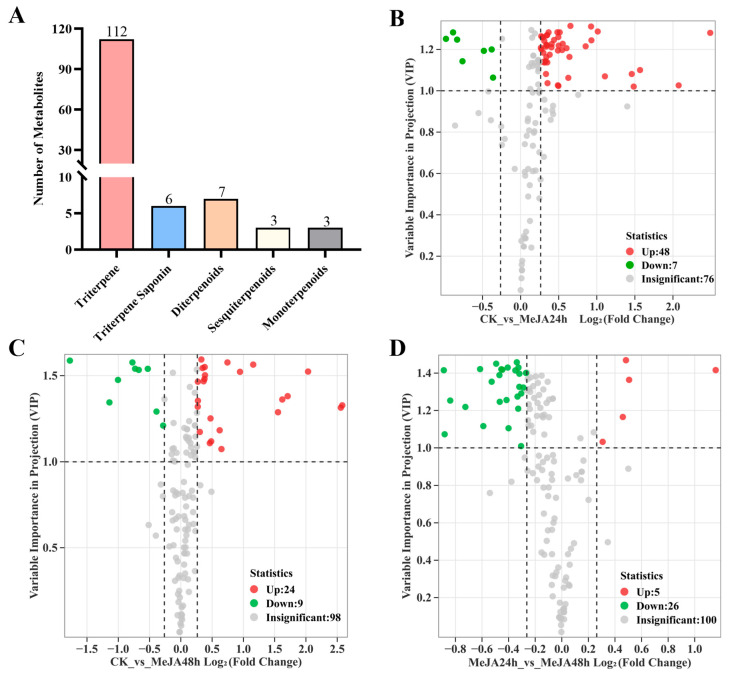
Widely targeted metabolome analysis of loquat callus under methyl jasmonate (MeJA) treatment. (**A**) Classes of detected terpenoid metabolites in loquat callus. (**B**–**D**) Volcano plots of differential metabolites (DEMs). (**B**) CK vs. MeJA_24 h. (**C**) CK vs. MeJA_48 h. (**D**) MeJA_24 h vs. MeJA_48 h. Each point represents a metabolite. The horizontal axis represents the fold change of metabolites between groups and the VIP value indicates a statistically significant difference.

A total of 131 terpenoid metabolites were detected in loquat callus, including 112 triterpenes, six triterpene saponins, seven diterpenoids, three sesquiterpenoids and three monoterpenoids (Figure 2A). According to thresholds of VIP ≥ 1 and fold changes of ≥1.20 or ≤0.83, 55 and 33 differential metabolites (DEMs) were screened between CK vs. MeJA_24 h (48 upregulated and seven downregulated) (Figure 2B) and CK vs. MeJA_48 h (24 upregulated and nine downregulated) (Figure 2C), and 31 DEMs were detected between MeJA_24 h and MeJA_48 h (five upregulated and 26 downregulated) (Figure 2D).

**Figure 3:**
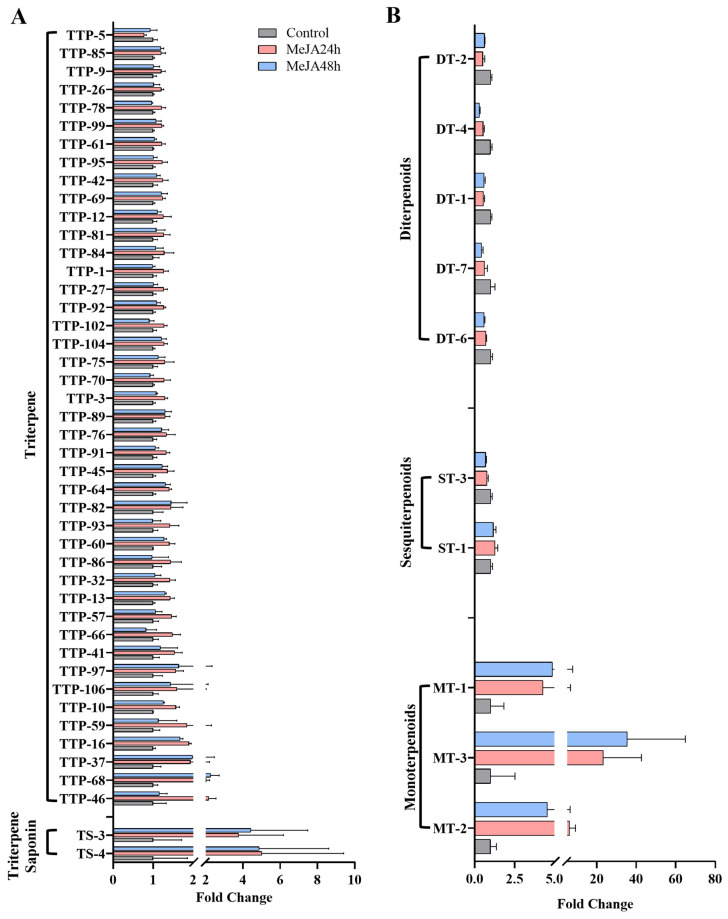
Fold changes of differential terpenoid metabolites in loquat callus under methyl jasmonate (MeJA) treatment. (**A**) Fold changes of triterpenes (TTPs) and triterpene saponins (TSs). (**B**) Fold changes of diterpenoids (DTs), sesquiterpenoids (STs) and monoterpenoids (MTs).

In CK vs. MeJA_24 h, most DEMs were triterpene compounds, accounting for 81.8% of all differential metabolites, whereas only five diterpenoids, two sesquiterpenoids and three monoterpenoids were identified (Table S1). Ursolic acid (TTP-85) was the most accumulated triterpene with a fold change of 1.20 and a VIP score of 1.21 at 24 h after MeJA treatment, and its levels were 20.2% higher than those of the control group. After 48 h of treatment, its abundance remained similar to that recorded within a 24 h period. Betulinic acid (TTP-76), the second most abundant compound (with a fold change of 1.32 and a VIP score of 1.21 at 24 h, compared to a fold change of 1.21 and VIP score of 1.35 at 48 h), displayed 1.32- and 1.21-fold increases at 24 and 48 h, respectively, in comparison with the untreated control (Figure 3A). 3,29-Dihydroxyolean-12-en-28-oic acid (TTP-46) and corosolic acid methyl ester (TTP-68) were the most significantly upregulated triterpenes after MeJA treatment was performed for 24 h and 48 h, respectively. Additionally, the amount of 3-O-p-coumaroyltormentic acid (TTP-106), a compound with anti-inflammatory activity, increased markedly at 24 h. Two triterpene saponin compounds [i.e., medicagenic acid-3-O-glucuronide-28-O-xylosyl(1,4)-[apiosyl(1,3)]-rhamnosyl(1,2)-arabinoside (TS-4) and medicagenic acid-3-O-glucuronide-28-O-xylosyl(1,4)-rhamnosyl(1,2)-arabinoside (TS-3)] also increased in both the MeJA_24 h and MeJA_48 h groups (Figure 3A). Among the diterpenoids, five compounds detected in loquat callus decreased significantly following MeJA treatment in both comparisons. Confertifoline (ST-1), a kind of sesquiterpenoid, increased in callus tissues when exposed to MeJA treatment for 24 h. All detected monoterpenoids, including genipin (MT-3), vomifoliol (MT-2) and perillyl alcohol (MT-1), were obviously upregulated under MeJA treatment (Figure 3B). These results indicated that MeJA treatment predominantly improved triterpene accumulation, whereas other terpenoid classes exhibited weaker or variable responses.

### 3.2. Transcriptome Sequencing and Differentially Expressed Gene Analysis in Loquat Callus Under MeJA Treatment

**Figure 4:**
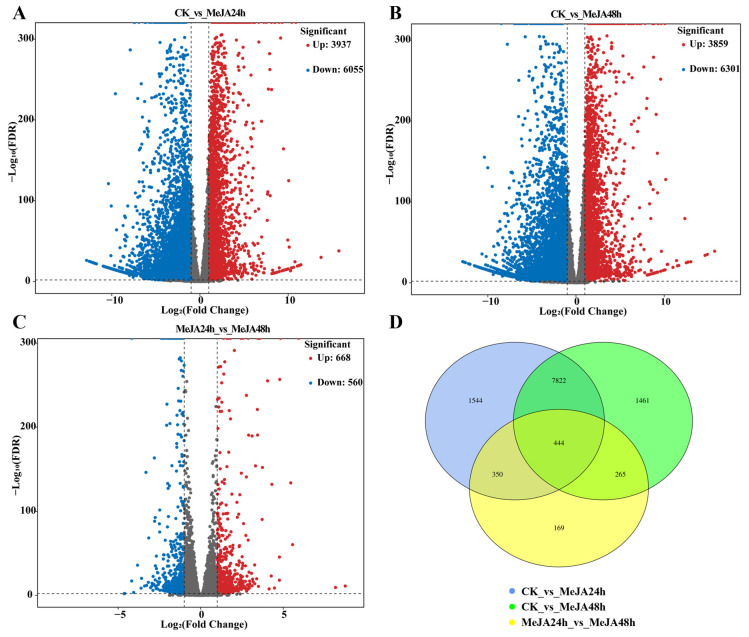
Differentially expressed genes (DEGs) screening and Venn diagram analysis. (**A**–**C**) The volcano plots for comparisons between MeJA-treated and control samples. (**D**) Venn diagram showing the DGEs distribution among different groups.

To further demonstrate the molecular basis of MeJA-induced terpenoid biosynthesis in loquat callus, transcriptomes were sequenced using an Illumina RNA-seq platform. The data output quality of the quantitative transcriptome sequencing is summarized in Table S2, including the total reads, mapped reads and Uniq Mapped reads, Multiple Map reads and the ratio of Q30. After quality trimming, a total of 67.18 Gb clean reads were obtained from nine samples. For all libraries, the percentage of Q30 was higher than 93.0%. A total of 9992 differentially expressed genes (DEGs) (3937 upregulated and 6055 downregulated) in CK vs. MeJA_24 h, 10,160 DEGs (3859 upregulated and 6301 downregulated) in CK vs. MeJA_48 h, and 1228 DEGs (668 upregulated and 560 downregulated) in MeJA_24 h vs. MeJA_48 h was identified, respectively (Figure 4A–C). A Venn diagram showed that 444 DEGs were shared among three comparisons (Figure 4D). Overall, MeJA treatment predominantly repressed transcription, with downregulated genes outnumbering upregulated genes in all comparisons.

**Figure 5:**
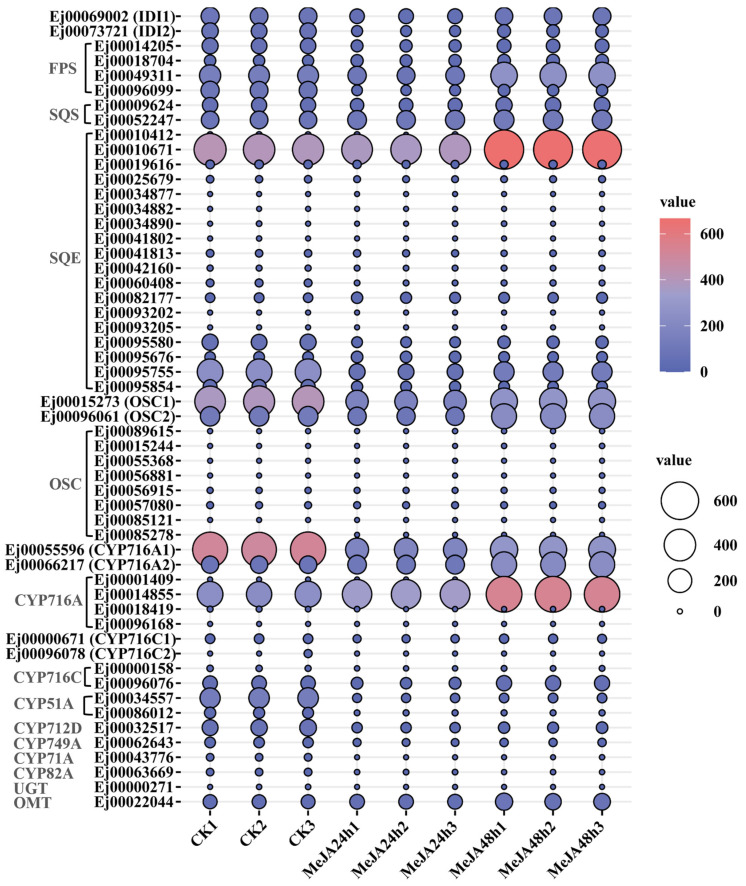
Expression profiling of genes involved in terpenoid biosynthesis in loquat callus under methyl jasmonate (MeJA) treatment. FPS: farnesyl pyrophosphate synthase; SQS: squalene synthase; SQE: squalene epoxidase; OSC: oxidosqualene cyclase; CYP: cytochrome P450 monooxygenases; UGT: glucosyltransferase; OMT: O-methyltransferase.

Based on gene annotation, a total of 55 candidate genes involved in terpenes biosynthesis were identified, of which 10 were upregulated and 13 were downregulated under MeJA treatment. OSC, a key rate-limiting enzyme in the downstream biosynthesis of triterpenes, catalyzes the cyclization of 2,3-oxidosqualene to produce sterols and triterpene precursors. CYP450 is responsible for structural modifications of the triterpene skeleton, thereby generating diverse triterpene compounds [12]. Previously, we characterized several loquat triterpene biosynthetic genes, such as *EjOSC1/2*, *EjCYP716A1/2* and *EjCYP716C1/2* [14]. *EjOSC2* (*Ej00096061*) and *EjCYP716A2* (*Ej00066217*) were induced by MeJA after 48 h of treatment. Additionally, a putative uncharacterized CYP716A subfamily gene (*Ej00014855*) also exhibited a markedly upregulated expression (Figure 5).

Farnesyl pyrophosphate synthase (FPS) and squalene epoxidase (SQE) are essential upstream enzymes participating in the production of 2,3-oxidosqualene, a common precursor of triterpenes [11]. Notably, six putative genes encoding these enzymes (*Ej00018704*, *Ej00049311*, *Ej00010412*, *Ej00010671*, *Ej00082177* and *Ej00095676*) were significantly upregulated after MeJA treatment, which was consistent with the increased terpenoid accumulation in MeJA-treated callus (Figure 5). Methylated triterpene acid derivatives have also been reported in loquat [32]. The expression of *Ej00022044*, annotated as a putative O-methyltransferase (OMT), also increased under MeJA treatment compared to the control group (Figure 5).

### 3.3. Differentially Expressed Genes Related to JA-Mediated Signaling Pathway and JA Response

**Figure 6:**
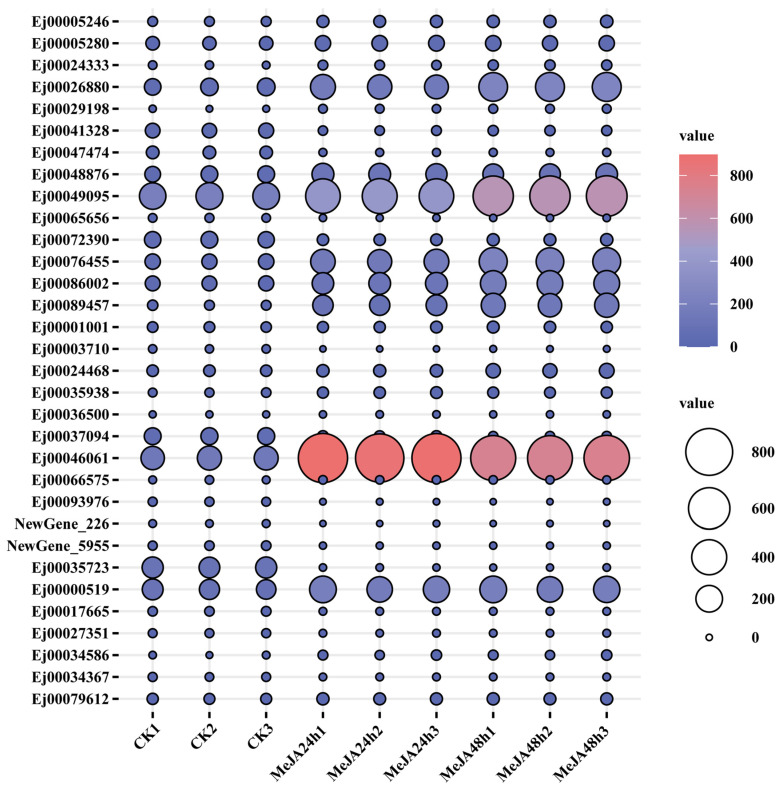
Expression profiling of genes related to jasmonic acid (JA) response in loquat callus under methyl jasmonate (MeJA) treatment.

Among DEGs, 32 genes associated with the jasmonic acid (JA)-mediated signaling pathway response exhibited significant expression change following MeJA treatment. Of these 32 genes, 19 genes were upregulated and 13 genes were downregulated. JASMONATE ZIM-DOMAIN (JAZ) proteins, key components of the JA signaling cascade, function as plant-specific negative regulators [33]. After MeJA treatment for 24 h and 48 h, *Ej00024333*, *Ej00026880* and *Ej00076455*, annotated as putative *JAZ* genes, were highly upregulated (Table S3 and Figure 6). TIFY is a novel plant-specific transcription factor involved in plant developmental processes, responses to hormones and signal transduction [34]. Nine genes were annotated as TIFY family. Among them, *Ej00048876*, *Ej00049095*, *Ej00086002*, *Ej00089457*, *Ej00000519* and *Ej00034586* were upregulated, whereas *Ej00047474*, *Ej00065656* and *Ej00072390* were downregulated in response to MeJA. Additionally, *Ej00046061* encoding a uncharacterized NAC domain-containing protein 2 participated in the JA response in plants was also significantly upregulated in MeJA-treated loquat callus (Table S3 and Figure 6).

### 3.4. Correlation Between Genes Involved in Terpenoid Biosynthesis and JA-Mediated Signaling Pathway and JA Response

**Figure 7:**
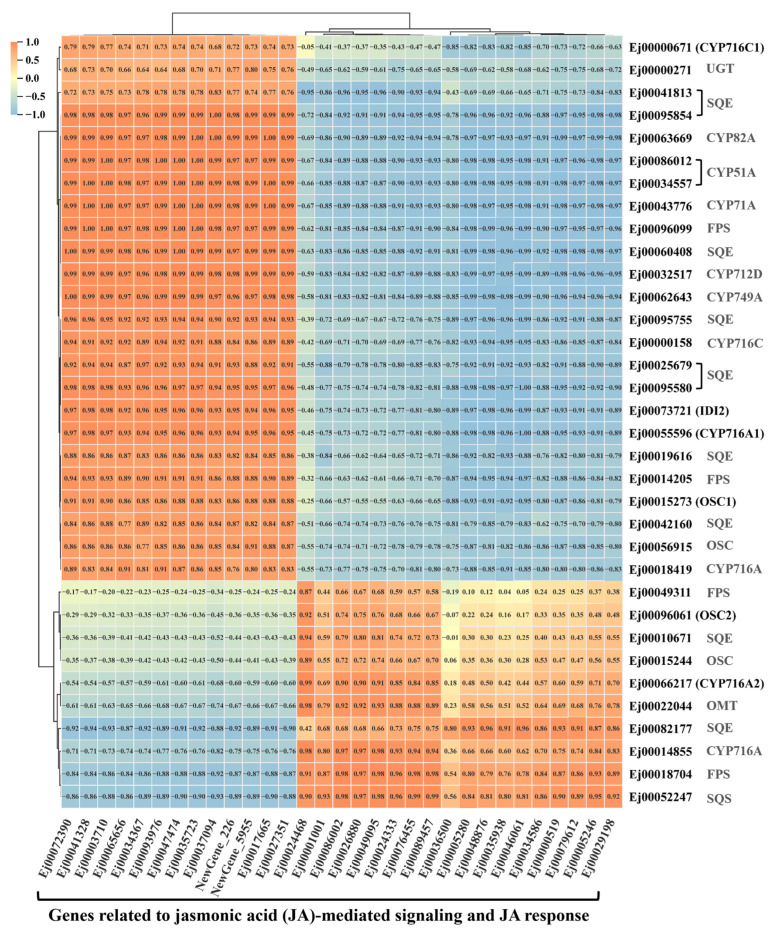
Heatmap representing the Pearson correlation coefficient between genes involved in triterpene biosynthesis and jasmonic acid (JA)-mediated signaling and JA response. FPS: farnesyl pyrophosphate synthase; SQS: squalene synthase; SQE: squalene epoxidase; OSC: oxidosqualene cyclase; CYP: cytochrome P450 monooxygenases; UGT: glucosyltransferase; OMT: O-methyltransferase. The orange zone indicates a positive correlation and the blue suggests a negative correlation. The numbers in each cell represent Pearson correlation coefficient values.

To evaluate the correlation between genes involved in terpenoid biosynthesis and JA-mediated signaling, as well as JA response, we performed a gene expression correlation analysis based on the Pearson correlation coefficient. The result showed that genes related to JA-mediated signaling, such as *Ej00072390*, *Ej00041328*, *Ej00065656*, *Ej00047474*, *Ej00017665*, *Ej00027351* and *Ej00035723*, as well as genes associated with JA response, including *Ej00003710*, *Ej00034367*, *Ej00093976* and *Ej00037094*, were positively correlated with the known triterpene biosynthetic genes *EjOSC1*, *EjCYP716A1* and *EjCYP716C1*, while they displayed a negative correlation with *EjOSC2* and *EjCYP716A2* (Figure 7). Furthermore, we also analyzed the cis-acting elements in promoters of terpenoid biosynthetic genes using PlantCARE. The TGACG-motif, a cis-acting regulatory element associated with MeJA-responsiveness, was identified in the promoters of *EjCYP716A1*, *EjCYP716A2* and *EjCYP716C2* (Table S4). Collectively, these findings revealed a contrasting correlation pattern among triterpene biosynthetic genes in response to JA signaling: *EjOSC1*, *EjCYP716A1* and *EjCYP716C1* were positively correlated with JA-related genes, whereas *EjOSC2* and *EjCYP716A2* exhibited negative correlations. These observations suggested potential transcriptional regulation between terpenoid biosynthesis and JA signaling in loquat, though further functional validation is needed to confirm these interactions.

## 4. Discussion

MeJA, the volatile methyl ester derivative of JA, can serve as an endogenous signaling molecule regulating plant growth and development, stress response, as well as secondary metabolites biosynthesis [19,35]. MeJA also affects fruit development, maturation and quality [36]. Exogenous MeJA treatment regulated ascorbic acid and glutathione metabolism, thereby enhancing the quality of loquat fruit [37]. Triterpenes, which are natural active compounds, accumulated in horticultural crops and other plants [12]. Cuticular wax triterpenes contributed to maintaining the postharvest storage quality of blueberry fruit by reducing water loss [38]. In loquat, triterpenes are the primary class of bioactive compounds, with 47 and 193 triterpenes identified in leaves and flowers, respectively [7,8]. Callus cultures induced from loquat axenic leaf could also accumulate large amounts of triterpenes [17]. In this study, we utilized a widely targeted metabolomics approach to detect terpenoids in loquat callus cultures that underwent MeJA treatment (Figure 1). A total of 131 terpenoid compounds were identified in loquat callus cultures, of which triterpenes accounted for 90.1% (Figure 2), suggesting that MeJA mainly activated triterpene biosynthesis in loquat callus. Triterpenes usually display antioxidant activity and effectively scavenge free radicals, which makes them valuable for developing functional food and nutraceutical ingredients [8]. Our results provide foundational knowledge for understanding how elicitor treatment can influence the accumulation of these health-associated compounds in loquat callus.

MeJA has been widely used as a major elicitor to enhance the production of secondary metabolites in various plant systems. In grape berry, exogenous MeJA promoted the accumulation of volatile monoterpenoid compounds [39]. MeJA treatment increased isoflavone production in soybean suspension cell cultures [20]. Asiaticoside, one of the principal triterpene saponins in leaves, was also induced by MeJA treatment in *Centella asiatica* (L.) hairy roots [40]. In agreement with these reports, MeJA induced terpenoid accumulation in loquat callus cultures. A total of 55 differential metabolites (DEMs) were detected in CK vs. MeJA_24 h (48 upregulated, seven downregulated), and 33 DEMs in CK vs. MeJA_48 h (24 upregulated and nine downregulated) (Figure 2). We also found that ursolic acid (TTP-85) was the most accumulated compound at 24 h. Betulinic acid (TTP-76), a natural lupane-type pentacyclic triterpene compound, exhibited the most abundant accumulation at 48 h (Figure 3). However, the magnitude of increase (e.g., approximately 20% or 1.3-fold) was moderate. The optimization of sample extraction and detection methods, as well as the expansion of the database, may increase the coverage of detectable metabolites.. Meanwhile, absolute quantification for these triterpene compounds was not performed in this study. Future studies employing targeted metabolomics with authentic standards would be valuable for assessing the biological or biotechnological relevance of the observed changes. Regarding the temporal differences in the accumulation of ursolic acid and betulinic acid between 24 h and 48 h treatments, we proposed that in loquat, the known OSC1/2 yielded a mixture of α-amyrin and β-amyrin at a ratio of 95:5 [14], which might lead to the rapid accumulation of ursolic acid through the flux of OSC1/2-CYP716A1/2 in loquat callus at 24 h. Meanwhile, ursolic acid could be furtherly catalyzed by EjCYP716C1/2 to produce corosolic acid. In contrast, the delayed predominance of betulinic acid at 48 h suggested that the lupeol synthase (catalyzing the precursor of betulinic acid) might be an unidentified EjOSCs with multifunctional catalytic activity, potentially accompanied by the formation of nonspecific products.

Exogenous MeJA treatment of in vitro cultures can induce the accumulation of bioactive compounds by regulating the transcript levels of genes encoding related biosynthetic enzymes [41]. MeJA-treated soybean cell cultures exhibited the enhanced isoflavone production and the upregulated expression of genes involved in the isoflavonoid biosynthesis pathway [20]. In hairy root cultures of *Solanum trilobatum* L., MeJA treatment improved the expression of *HMGR* (HMGCoA reductase) and promoted solasodine production [42]. Similarly, in ginseng hairy root cultures, MeJA upregulated *PgSS* (squalene synthase), *PgSE* (squalene epoxidase) and *PNA* (dammarenediol synthase-II) transcripts, together with the elevated ginsenosides levels [43]. Consistent with these previous findings, our transcriptome analysis revealed that MeJA significantly upregulated the structural genes involved in triterpene biosynthesis in loquat callus, including the identified *EjOSC2* and *EjCYP716A2*, as well as putative *CYP716A*, *FPS*, *SQE* and *OMT* genes (Figure 5), which contributed to terpenoid accumulation in MeJA-treated loquat callus. However, the upregulation of these key genes was not verified by qRT-PCR. Meanwhile, enzyme activities and metabolic fluxes analysis were not directly detected in this study. Therefore, further investigations involving qRT-PCR validation, enzymatic assays or metabolic flux analysis will be important to confirm the functional consequences of transcriptional changes.

MeJA responds to secondary metabolite pathways in plants, which is correlated with the JA signaling pathways [44]. In grape berry skin, MeJA enhanced the contents of volatile monoterpenoids, and upregulated the transcripts of genes associated with JA signal pathway, including *JAZ* genes [39]. In *Hevea brasiliensis*, the genes encoding JA signaling components such as *JAZ* genes were found to be differentially expressed under MeJA treatment, accompanied by the upregulated expression of key genes in rubber biosynthesis pathway [45]. Similar results were observed in our study, MeJA also induced the upregulation of putative JA signaling genes (such as *JAZ* and *TIFY*) in loquat callus (Table S3 and Figure 6). In addition, most triterpene biosynthetic genes were correlated with genes associated with JA-mediated signaling and JA response (Figure 7). An interesting observation was the differential correlation of JA signaling genes with *EjOSC1* and *EjOSC2*. This opposite correlations might reflect sub-functionalization at the expression level, where the two isoforms (*EjOSC1* and *EjOSC2*) might be deployed under different physiological conditions or in different tissues to ensure robust triterpene production. Promoter analysis further showed that the promoters of *EjCYP716A1*, *EjCYP716A2* and *EjCYP716C2* contained the TGACG-motif, a cis-acting regulatory element related to MeJA-responsiveness (Table S4). Taken together, we proposed a possible working model in which MeJA induced the expression of the JA signaling cascade, including JA-responsive regulators *JAZ* and *TIFY* genes. These factors potentially bind to the TGACG motif in the promoters of triterpene biosynthetic genes, modulating their expression and ultimately leading to triterpenoid accumulation in loquat callus. This proposed regulatory relationship needs to be further validated by additional experiments (e.g., gene overexpression, gene silencing, or promoter-reporter assays).

## 5. Conclusions

In summary, our study provided the first integrated metabolomic and transcriptomic analysis of loquat callus response to MeJA. By combining widely targeted metabolomics with transcriptome profiling, we concluded that MeJA modulated the expression of triterpene biosynthetic genes and JA signaling components, leading to differential triterpenoid accumulation. These findings not only contribute to our understanding of the regulation of triterpene metabolism in loquat, but also provide possibilities for developing plant callus culture strategies for improved biosynthesis of bioactive triterpenoids with nutraceutical potential.

## Data Availability

The original contributions presented in this study are included in the article/Supplementary Materials. Further inquiries can be directed to the corresponding authors.

## Supplementary Materials

The following supporting information can be downloaded at https://www.mdpi.com/article/10.3390/foods15061078/s1, Table S1: All detected terpenoid metabolites in loquat callus under methyl jasmonate (MeJA) treatment; Table S2: Summary of data quality in transcriptome sequencing; Table S3: Differentially expressed genes in loquat callus under methyl jasmonate (MeJA) treatment; Table S4: The analysis of cis-acting elements in the promoters of triterpene biosynthetic genes using PlantCARE.
