# Supplementary material for: Exogenous Methyl Jasmonate Promotes Triterpene Accumulation in Loquat Callus
Source: Foods. 2026 Mar 19;15(6):1078. doi: 10.3390/foods15061078 (PMC13024789; doi:10.3390/foods15061078)
Supplement: Supplementary file 1 [file foods-15-01078-s001.zip › Table S2.pdf]

Supplementary Table S2 Summary of data quality in transcriptome sequencing.

Sample	Total reads	mapped reads	Uniq Mapped reads	Multiple Map Reads	Reads Map to '+'	Reads Map to '-'	%≥Q30
**CK1**	39,733,232	36,592,951 (92.10%)	34,341,813 (86.43%)	2,251,138 (5.67%)	20,800,625 (52.35%)	20,819,707 (52.40%)	94.25%
**CK2**	47,325,860	43,737,512 (92.42%)	41,072,628 (86.79%)	2,664,884 (5.63%)	24,758,441 (52.31%)	24,781,137 (52.36%)	93.56%
**CK3**	43,169,392	39,865,815 (92.35%)	37,416,475 (86.67%)	2,449,340 (5.67%)	22,600,665 (52.35%)	22,619,555 (52.40%)	94.11%
**MeJA-24h1**	71,989,018	67,045,065 (93.13%)	60,869,040 (84.55%)	6,176,025 (8.58%)	40,466,096 (56.21%)	40,472,391 (56.22%)	93.88%
**MeJA-24h2**	47,841,098	44,633,116 (93.29%)	39,710,203 (83.00%)	4,922,913 (10.29%)	28,531,531 (59.64%)	28,551,245 (59.68%)	94.17%
**MeJA-24h3**	49,920,138	46,291,026 (92.73%)	42,123,867 (84.38%)	4,167,159 (8.35%)	27,782,279 (55.65%)	27,785,843 (55.66%)	94.14%
**MeJA-48h1**	50,260,148	46,711,292 (92.94%)	42,217,657 (84.00%)	4,493,635 (8.94%)	28,408,891 (56.52%)	28,401,606 (56.51%)	94.01%
**MeJA-48h2**	48,515,388	44,928,550 (92.61%)	40,713,369 (83.92%)	4,215,181 (8.69%)	27,177,553 (56.02%)	27,195,542 (56.06%)	93.69%
**MeJA-48h3**	52,151,188	48,638,863 (93.27%)	43,868,763 (84.12%)	4,770,100 (9.15%)	29,736,648 (57.02%)	29,717,552 (56.98%)	94.45%

(1) Total Reads: The number of Clean Reads, counted on a single end;

(2) Mapped Reads: The number of Reads that are mapped to the reference genome and the percentage of them in Clean Reads;

(3) Uniq Mapped Reads: The number of Reads that are mapped to a unique position on the reference genome and the percentage of them in Clean Reads;

(4) Multiple Map Reads: The number of Reads that are mapped to multiple positions on the reference genome and the percentage of them in Clean Reads;

(5) Reads Map to '+': The number of Reads that are mapped to the positive strand of the reference genome and the percentage of them in Clean Reads;

(6) Reads Map to '-': The number of Reads that are mapped to the negative strand of the reference genome and the percentage of them in Clean Reads;

(7) %≥Q30：The percentages of the bases with clean data quality values larger than 30 in the total reads obtained by sequencing.
